# Transmission of *Fusarium boothii* Mycovirus via Protoplast Fusion Causes Hypovirulence in Other Phytopathogenic Fungi

**DOI:** 10.1371/journal.pone.0021629

**Published:** 2011-06-29

**Authors:** Kyung-Mi Lee, Jisuk Yu, Moonil Son, Yin-Won Lee, Kook-Hyung Kim

**Affiliations:** Department of Agricultural Biotechnology and Center for Fungal Pathogenesis, Seoul National University, Seoul, Korea; Institute of Developmental Biology and Cancer Research, France

## Abstract

There is increasing concern regarding the use of fungicides to control plant diseases, whereby interest has increased in the biological control of phytopathogenic fungi by the application of hypovirulent mycoviruses as a possible alternative to fungicides. Transmission of hypovirulence-associated double-stranded RNA (dsRNA) viruses between mycelia, however, is prevented by the vegetative incompatibility barrier that often exists between different species or strains of filamentous fungi. We determined whether protoplast fusion could be used to transmit FgV1-DK21 virus, which is associated with hypovirulence on *F. boothii* (formerly *F. graminearum* strain DK21), to *F. graminearum*, *F. asiaticum*, *F. oxysporum* f. sp. *lycopersici*, and *Cryphonectria parasitica*. Relative to virus-free strains, the FgV1-DK21 recipient strains had reduced growth rates, altered pigmentation, and reduced virulence. These results indicate that protoplast fusion can be used to introduce FgV1-DK21 dsRNA into other *Fusarium* species and into *C. parasitica* and that FgV1-DK21 can be used as a hypovirulence factor and thus as a biological control agent.

## Introduction

Although fungicides successfully control many diseases caused by plant-pathogenic fungi, fungal pathogens remain a major source of plant disease. Because of the development of fungicide-resistant strains, and increasing public concern regarding environmental and food safety, there is renewed interest in biological control based on application of hypovirulent mycoviruses.

The potential of mycoviruses for managing plant-pathogenic fungi was first demonstrated for *Cryphonectria parasitica* [1]. The success of biological control with hypoviruses depends on their ability to reduce the virulence (to induce hypovirulence) of the target fungus. Hypoviruses can be transmitted from a hypovirulent strain to a virulent fungal strain by hyphal fusion (anastomosis) when the two strains are vegetatively compatible, but hypoviruses cannot be transmitted when applied by extracellular routes [2], [3]. Because only closely related fungal strains are vegetatively compatible, vegetative incompatibility among many fungal species in agricultural ecosystems is a major barrier to the use of hypoviruses as biological control agents [4], [5].

Double-stranded RNA mycoviruses have been described in yeasts, mushrooms, and filamentous fungi [6], [7], [8]. They are classified into five families based on virus structure and genome composition [9], but some are still unassigned to a genus or in some cases to a family. There is increasing evidence that mycoviruses reduce the growth and pathogenicity of fungal plant pathogens. As noted above, a virulence-attenuating dsRNA molecule has been described in *C. parasitica*, and five related mycoviruses have been completely sequenced [9]. Among them, *Cryphonectria hypovirus 1* (CHV1) was successfully used as a biological control agent of *C. parasitica* in Europe, i.e., CHV1-infected strains exhibited reduced virulence, reduced asexual and sexual sporulation, and reduced pigment production. CHV1 was unsuccessful as a biological control agent in North America, however, because the host fungus in North America has multiple vegetative compatibility groups (VCGs) that limit the spread of the virus [10].

The failure of mycovirus transmission caused by vegetative incompatibility can be overcome in the laboratory by using protoplast fusion [11]. Transmission of dsRNA mycoviruses via protoplast fusion has been reported in plant-pathogenic fungi including *Aspergillus* [12], *F. poae* [13], and *Rosellinia necatrix* [14].

We previously isolated the FgV1-DK21 virus from strain DK21 [15]. According to genealogical concordance phylogenetic species recognition (GCPSR), the *F. graminearum* species complex (*Fg* complex) comprises 13 phylogenetically distinct species based on DNA sequences from 13 independent genetic loci [16], [17]. Strain DK21 was evaluated by GCPSR using DNA sequences from selected genes and it was identified as *F. boothii* (Figure S1). FgV1-DK21 reduces the mycelial growth of *F. boothii*, increases its pigmentation, and reduces its virulence on wheat [15]. The 6,621 nucleotide-coding strand is polyadenylated and contains four open reading frames (ORFs 1 to 4) [18]. Pairwise sequence comparisons of the nucleotide and deduced amino acid sequences of ORFs 2 through 4 revealed no close relationships to other protein sequences currently available in GenBank while a phylogenetic analysis of the deduced amino acid sequence of ORF1, which encodes a putative RNA-dependent RNA polymerase (RdRp), and those of other mycoviruses revealed that this organism forms a distinct virus clade with some hypoviruses and is more distantly related to other mycoviruses [18]. While FgV1-DK21 does not encode a coat protein, the genome organization and accumulation of at least two subgenomic RNAs (sgRNAs) indicate that FgV1-DK21 belongs to a new, as yet unassigned genus of mycoviruses [18].

In this report, we present evidence that protoplast fusion can be used to expand hypovirus host range and to study hypovirus-mediated alterations in new fungal hosts. The results of this study indicate that protoplast fusion can overcome the barriers to transmission caused by genetic diversity and multiple VCGs and thus will extend persistent and transmissible system with application of FgV1-DK21 for fungal disease control.

## Results

### Effect of FgV1-DK21 dsRNA on colony morphology and mycelial growth

**Figure 1:**
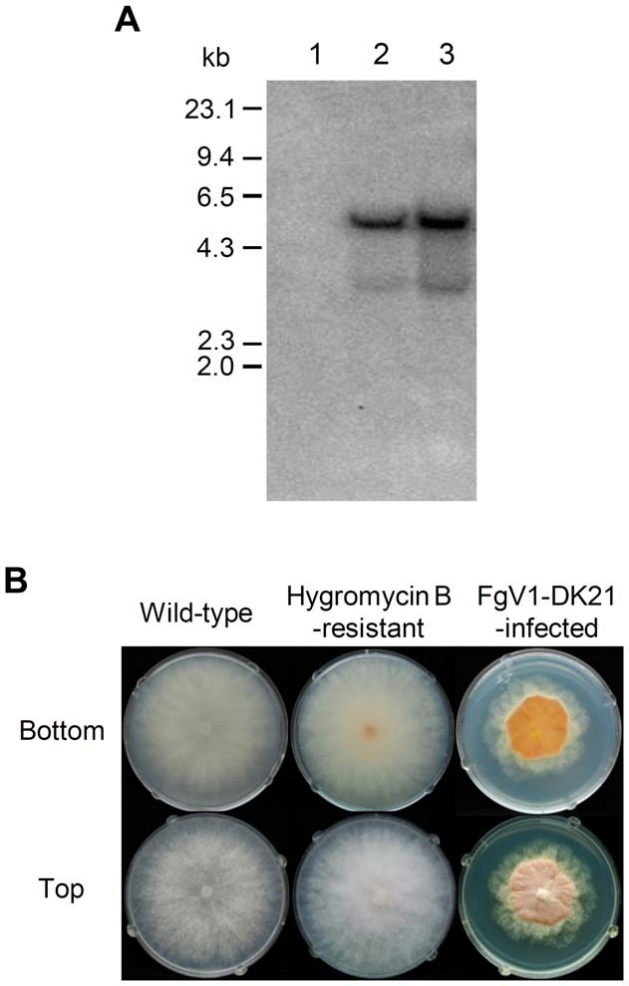
Transformation of *F. graminearum* with a hygromycin B resistance gene. (A) Southern blot hybridization of *Kpn* I*-*digested genomic DNAs. Hygromycin B-resistant transformants were obtained by transforming the fungal protoplasts with the plasmid pUCH1. The probe used was *Eco*R I and *Hind* III fragment (1.4 kb) from pUCH1 bearing the *hygB* structural gene. Lane M, λ DNA-*Hind* III digested DNA marker; lane 1, wild-type; lane 2, hygromycin B-resistant strain; lane 3, virus-infected strain (protoplast fusant). (B) Photograph of fungal colonies 4 days after inoculation.

**Figure 2:**
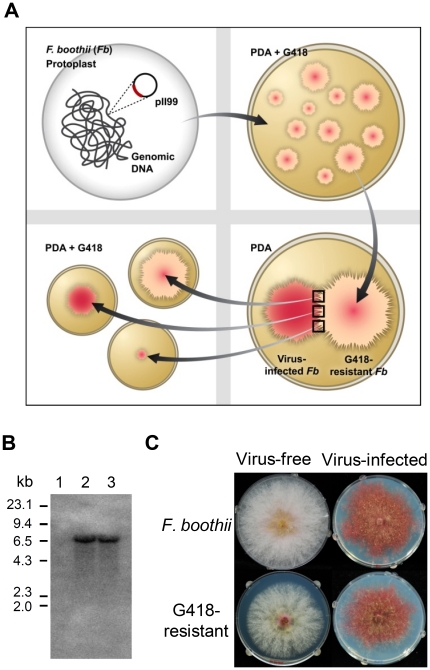
Construction of virus-infected G418-resistant mutant. (A) Strategy of construction of G418-resistant mutant. (B) Southern blotting of *Kpn* I and *Spe* I -digested genomic DNAs, hybridized with a 1.9 kb geneticin probe. Lane M, λ DNA-*Hind* III digested DNA marker; lane 1, wild-type; lane 2, G418-resistant strain; lane 3, virus-infected strain (G418-selected). (C) Colony morphology of virus-free and -infected of wild-type and G418-resistant strains 5 days after inoculation.

We first determined whether FgV1-DK21 can overcome the VCG barrier in other *Fusarium* species. One strain each of two species within the *Fg* complex [*F. asiaticum* [19] and *F. graminearum* [20]], and one strain of *F. oxysporum* f. sp. *lycopersici* (outgroup) were chosen (Table 1). To improve screening efficiency of fused protoplasts, virus-free recipients and the virus-infected donors were transformed with hygromycin B- and geneticin-resistance genes, respectively (Figures 1 and 2). After equal volumes of the two protoplast suspensions (1×10^6^ protoplasts/ml) were fused by the 60% polyethylene glycol (PEG 3350)-mediated method, virus-infected strains were finally selected on hygromycin B-containing PDA (see *Materials and Methods*). Several virus-infected strains (2, 8, and 9) were selected for *F. asiaticum*, *F. graminearum*, and *F. oxysporum* f. sp. *lycopersici*, respectively (data not shown).

**Table 1:** Strains included in this study.

Taxon	Characteristics^a^	Reproduction	Reference
*F. boothii* (*Fb*)	Strain DK21; vius-free and virus-infected (*Gen^R^*)	Homothallic	15
*F. asiaticum* (*Fa*)	Strain 88-1 (*HygB^R^*)	Homothallic	19
*F. graminearum* (*Fg*)	Strain DK3; virus-free (*Hyg^R^*)	Homothallic	20
*F. oxysporum* f. sp. *lycopersici* (*Fo*)	*HygB^R^*	Asexual	This study
*Cryphonectria parasitica* (*Cp*)	*HygB^R^*	Heterothallic	This study

a *HygB^R^*, resistant to hygromycin B; *Gen^R^*, resistant to G418. Virus-free strains derived from strain DK21 and DK3 were obtained by single conidial isolation.

**Figure 3:**
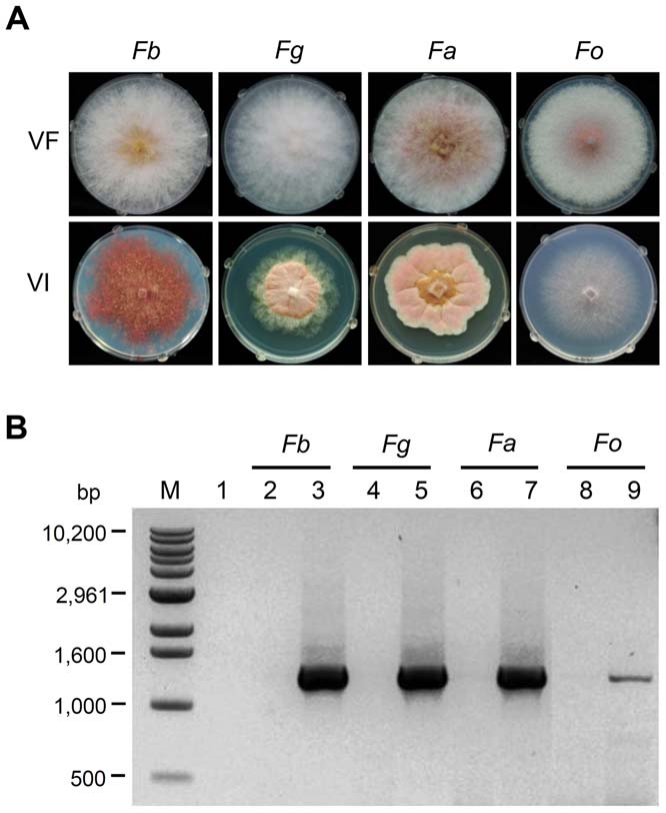
Phenotype of fungal colonies and reverse-transcription polymerase chain reaction (RT-PCR) analysis of *Fusarium* strains. *Fb* = *F. boothii*; *Fg* = *F. graminearum*; *Fa* = *F. asiaticum*; *Fo* = *F. oxysporum* f. sp. *lycopersici*. (A) Colony morphology of virus-free (VF) and virus-infected (VI) strains by protoplast fusion. (B) RT-PCR analysis of dsRNA in fungal strains. Lane M, 1-kb ladder DNA size marker; lane 1, negative control (no DNA template); lanes 2, 4, 6, and 8, virus-free strains; lanes 3, 5, 7, and 9, virus-infected strains. Presence of viral dsRNA was confirmed by RT-PCR amplification with a primer pair designed from the RdRp sequence of FgV1-DK21.

**Figure 4:**
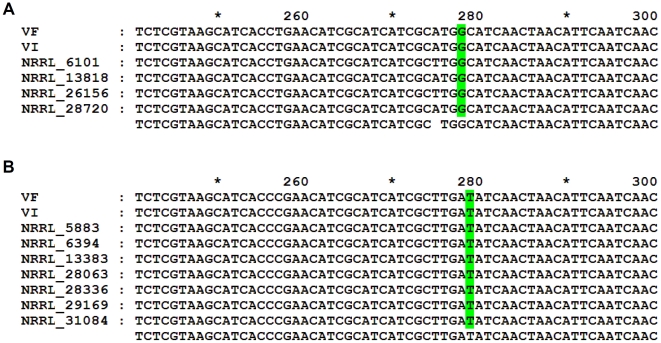
Alignments of histone H3 sequences from *F. asiaticum* (A) and *F. graminearum* (B) strains. VF = virus-free; VI = virus-infected. The fixed nucleotide characters are shaded in green. The presence of nucleotides G (position 278) and T (position 279) is differentially fixed for *F. asiaticum* and *F. graminearum*, respectively. GenBank accession numbers of nucleotide sequences used are as follows: NRRL 6101 (AY452820.1), NRRL 13818 (AY452821.1), NRRL 26156 (AY452843.1), NRRL 28720 (AY452844.1), NRRL 5883 (AY452815.1), NRRL 6394 (AY452817.1), NRRL 13383 (AY452819.1), NRRL 28063 (AY452816.1), NRRL 28336 (AY452818.1), NRRL 29169 (AY452836.1), NRRL 31084 (AY452852.1).

The phenotypic changes of virus-infected strains of *F. asiaticum* and *F. graminearum* were similar to those of strain DK21. Like strain DK21, FgV1-DK21 recipient strains of *F. asiaticum* and *F. graminearum* had reduced growth rates and increased pigmentation relative to virus-free strains (Figure 3A). In contrast, only slight morphological alterations were evident in the virus-infected *F. oxysporum* f. sp. *lycopersici* strain when growing on PDA (Figure 3A). However, the FgV1-DK21-infected strains of *Fusarium* species produced less aerial hyphae than the virus-free strains. FgV1-DK21 dsRNAs were detected in *F. graminearum* and *F. asaticum* strains, but not in *F. oxysporum* f. sp. *lycopersici* when extracted total RNAs were separated on agarose gel (Figure S2A). PCR amplified much more viral dsRNA in the virus-infected strains, *F. asiaticum* and *F. graminearum*, than in the virus-infected strain of *F. oxysporum* f. sp. *lycopersici* (Figures 4B and S2C). We also sequenced DNA from portions of translation elongation factor 1α (TEF) gene and/or histone H3 gene to determine whether dsRNA of strain DK21 was transferred into the desired recipient strain. The TEF and histone H3 genes have been used as phylogenetic markers to investigate species limits in *Fusarium* [16], [21]. As a consequence, the fixed nucleotide characters found in the virus-free strains of *F. asiaticum* (Histone H3 position 278; G) and *F. graminearum* (Histone H3 position 279; T) were also present in each virus-infected strain (Figure 4). Although the results indicate that dsRNA of strain DK21 was transferred into the recipient strains, it is unclear whether the altered phenotypes of recipient strains were the result of virus transmission or protoplast fusion because the recipient strains were different in terms of their morphology and in pathogenicity [22]. To address this concern, we analyzed DNA polymorphism between uninfected and virus-infected strains using amplified fragment length polymorphism (AFLP) profiling. Because the AFLP technique is based on the selective PCR amplification of restriction fragments from a total digest of genomic DNA, it will generate fingerprints of any DNA regardless of the origin or complexity and thus reflect true DNA polymorphisms [23]. The genomic DNAs from virus-free and virus-infected strains were digested with *Eco*R I and *Mse* I for AFLP analysis (Text S1). The digested DNAs were ligated with two adapters and amplified by PCR using specific oligonucleotide primers. Identical AFLP profiles were observed when we compared the DNA fingerprints among both uninfected and virus-infected samples (Figure S3A), indicating that the recipient strains screened from fused protoplasts were not substantially affected by protoplast fusion.

### Hypovirulence of FgV1-DK21 in other *Fusarium* species

**Figure 5:**
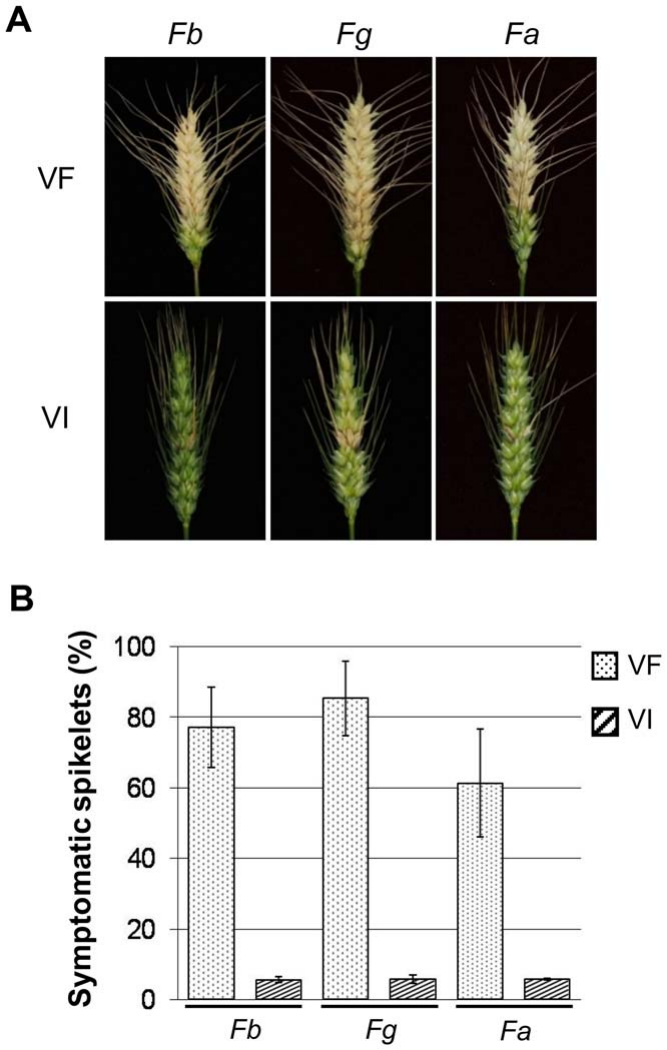
Disease symptoms in wheat head spikelets inoculated with fungal strains belonging to the *Fusarium graminearum* species complex. Conidial suspensions of each strain either uninfected or infected with FgV1-DK21 were used to inoculate wheat plants. Error bars indicate standard deviation.

Based on the previous observation that the virulence of strain DK21 was significantly lower than that of the virus-free strain [15], we hypothesized that FgV1-DK21 dsRNA might also contribute to the hypovirulence in other *Fusarium* species. To explore this possibility, wheat head florets were inoculated with conidial suspensions of virus-free or virus-infected strains of *F. asiaticum* and *F. graminearum* at early–mid anthesis. Head blight was more severe on wheat plants inoculated with virus-free strains than with virus-infected strains of *F. asiaticum* and *F. graminearum* (Figure 5).

**Figure 6:**
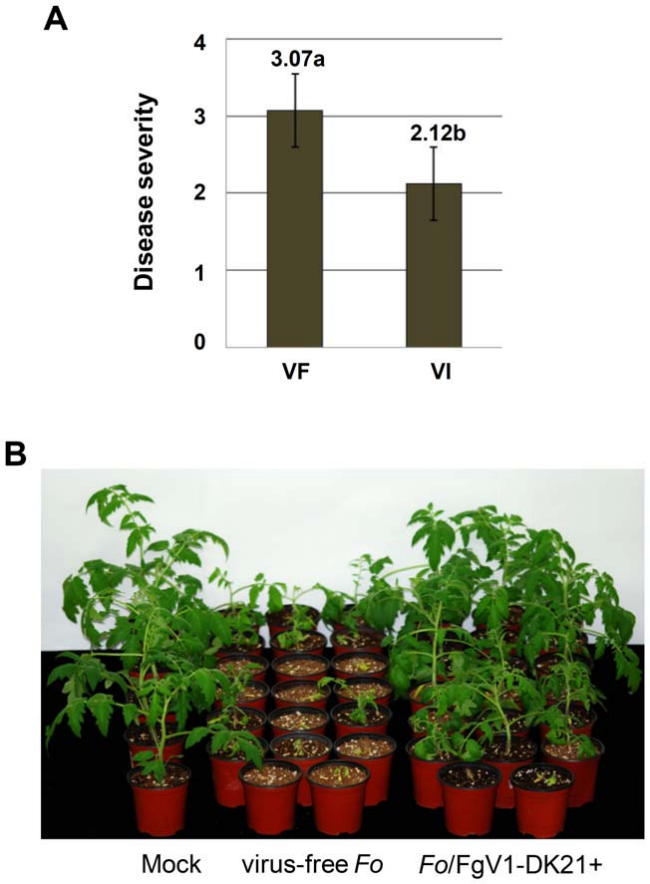
Virulence of virus-free (VF) and virus-infected (VI) strains of *F. oxysporum* f. sp. *lycopersici* on tomato seedlings. (A) Disease severity caused by fungal strains 3 weeks after inoculation. Disease index was scored on a scale of 0–4: 0, healthy plant; 1, slightly swollen and/or bent hypocotyl; 2, one brown vascular bundle in hypocotyl; 3, at least two brown vascular bundles and/or severe growth distortion (asymmetric development); 4, at least three brown vascular bundles and/or very small, wilted plant (or dead). Data were analyzed by the General Lineal Model (GLM) using PASW statistics 18.0 for Windows software (SPSS Inc.). Error bars indicate standard deviation. Different letters above the bars indicate significant differences at p≤0.05. (B) Photograph of tomato seedlings 4 weeks after inoculation.

For virulence assays with *F. oxysporum* f. sp. *lycopersici*, tomato seedlings growing in pots and at the four-leaf stage were inoculated with virus-free and virus-infected *F. oxysporum* f. sp. *lycopersici* strains by the root-dip method. At 3 weeks post-inoculation, seedlings were removed from the pots and their roots were observed for symptoms. Fusarium wilt had developed to the stem base in symptomatic seedlings, and the virus-infected strains were less virulent than the virus-free strains (Figure 6A). At 4 weeks post inoculation, 46 of 60 plants (76.7%) inoculated with the virus-free strains were dead and 43 of 60 plants (71.7%) inoculated with virus-infected strains remained alive (Figure 6B).

### Transmission of FgV1-DK21 dsRNA from strain DK21 to *C. parasitica*

**Figure 7:**
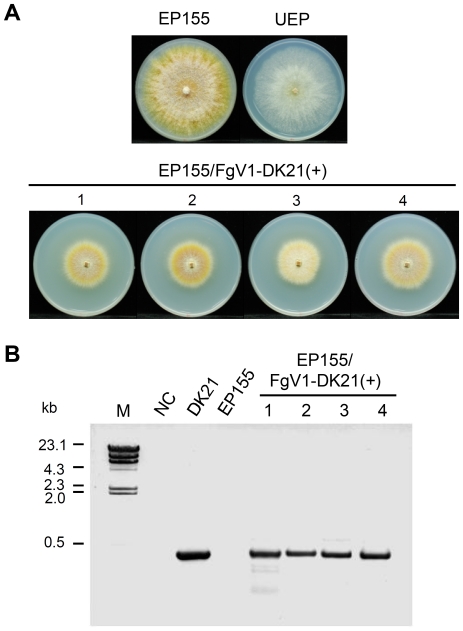
Phenotype and growth rate of *C. parasitica* as affected by transmission of dsRNA. Colony morphology (A) and RT-PCR analysis (B). EP155 (hygromycin B-resistant mutant), virus-free *C. parasitica* strain; UEP, EP155 infected with CHV1; 1 to 4, EP155 infected with FgV1-DK21. Lane M, λ DNA-*Hind* III digested DNA marker; NC, no DNA template.

**Figure 8:**
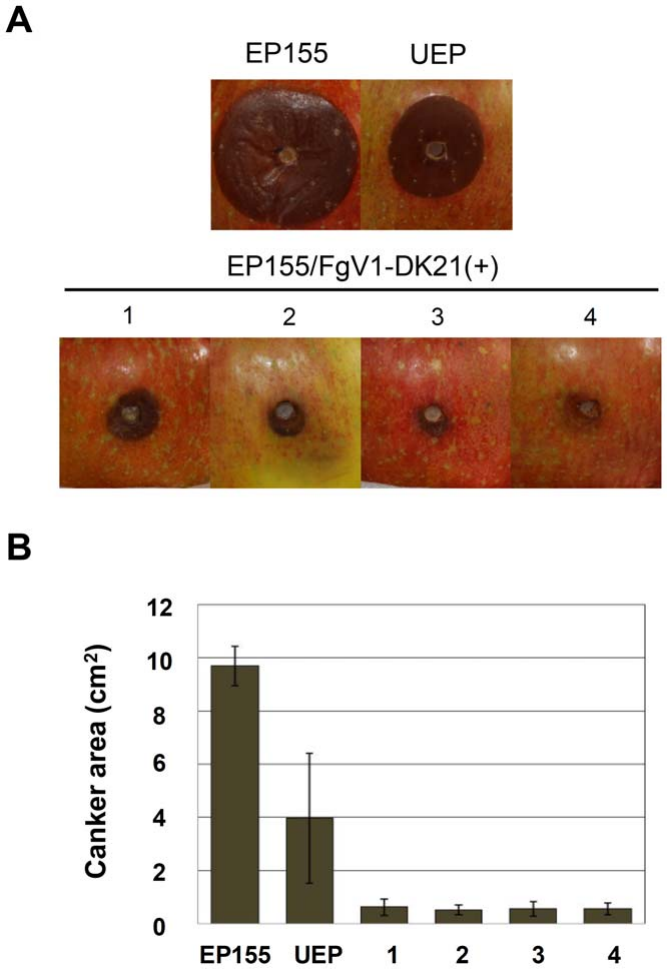
Virulence of *C. parasitica* strains on apples 3 weeks after inoculation. (A) Cankers induced by each strain on apples. Apples were inoculated with fresh cultures of strain EP155 either uninfected (EP155) or infected with CHV1 (UEP) or infected with FgV1-DK21 (1 to 4). (B) Size of cankers produced by the fungal strains. Error bars indicate standard deviation.

We also tested whether protoplast fusion can be used to introduce FgV1-DK21 dsRNA into a filamentous fungus of a different genus. *Cryphonectria parasitica* and associated mycoviruses provide a good model for studying virus/virus and virus/host interactions. For this reason, *C. parasitica* was subjected to protoplast fusion and evaluated as a potential host of FgV1-DK21. *Cryphonectria parasitica* strain EP155 was transformed with the hygromycin B resistance gene and fused as a recipient strain with strain DK21 (virus donor) by protoplast fusion. Four strains produced by the fusion procedure were selected and compared with virus-free EP155 and CHV1-infected EP155. CHV1-infected colonies (UEP) were smaller than virus-free colonies and lacked the orange pigment of virus-free colonies (Figure 7A). Colonies infected by FgV1-DK21 retained the orange color but were much smaller than virus-free EP155 or CHV1-infected EP155 colonies (Figure 7A). FgV1-DK21 was detected in virus-infected colonies by RT-PCR (Figures 7B and S2C). We also identified parallel bands among uninfected or virus-infected strains from AFLP profiling (Figure S3B) indicating that the uninfected and virus-infected strains of EP155 had not been altered significantly by the fusion process. In a virulence test with apples, the areas of lesions caused by virus-free EP155, the CHV1-infected strain, and FgV1-DK21-infected strains were approximately 10, 4, and 0.5 cm^2^, respectively (Figure 8A and B).

## Discussion

Here, we demonstrate that the FgV1-DK21 functions as a hypovirulence factor, thereby leading to morphological changes and hypovirulence. Our studies also present evidence that the protoplast fusion system can be used as a means for studying hypovirus-mediated alterations and strain development potential. Protoplast fusion technology has frequently been used for the genetic manipulation of fungi (for establishing heterokaryons) when few molecular genetic tools are available [24], [25]. By facilitating the discovery of new intermediates and hybrid antibiotics with beneficial properties [26], the large-scale enhancement of metabolite yields [27], and the construction of starch-utilizing strains [28], protoplast fusion has shown great potential for industrial applications. The present study, however, required that the genetic background of the recipient isolates be maintained when protoplast fusion was used to transfer hypovirulent dsRNA mycovirus to the recipients. Protoplast fusion with specific selection marker(s) has proven to be a novel approach by which potential strains with desirable properties could be obtained with minimal disturbance to their genetic background and physiology [29]. The genetic variability of recipient isolates was studied by sequencing the TEF and histone H3 gene region and by AFLP analysis. Banding patterns from the PCR-based AFLP method using total DNA from the dsRNA-free and dsRNA-containing recipient isolates were almost identical in most, if not all, tested samples (Figure S3). These observations suggest that the genetic background of the fungi suffered minimal disturbance from the fusion procedure and further confirmed the successful transmission of a dsRNA between the donor and the recipient isolates. Altogether, the present results demonstrate that protoplast fusion can be used to transmit FgV1-DK21 dsRNA both interspecifically and intergenerically.

Virulence-attenuating mycoviruses have been described in many plant-pathogenic fungi [9], however, few successful applications have been reported. Given that these mycoviruses are primarily transmitted horizontally via hyphal anastomosis or vertically from mycelium to spores, vegetative incompatibility within fungal species and the varying degree of vertical transmission efficiencies [10] are major barriers to their use as biological control agents. Consequently, the use of hypoviruses to control fungal diseases is restricted more by their limited natural transmission and/or lack of interspecies transmission than the availability of hypovirulence-associated mycoviruses. The development of efficient transmission and delivery methods might accelerate the use of hypovirulence-associated mycoviruses as biological control agents. In this study, transmitted virus (FgV1-DK21) replicated in the new hosts, though protoplast regeneration capacity was different between the strains tested. It is worth noting that hypovirulence induced in *C. parasitica* and measured in apple inoculations was greater with FgV1-DK21 dsRNA than with CHV1 (Figure 7A). The mechanisms by which the FgV1-DK21 causes hypovirulence to their hosts are unknown. How the FgV1-DK21 affects fungal physiology and virulence will have implications for other phytopathogenic fungi. For example, putative transcription factor PRO1 is down-regulated by *C. parasitica* strains infected with different hypoviruses and required for female fertility, asexual spore development, and stable maintenance of viral infection [30]. In light of these, comparative analysis of background- or mycovirus-related transcriptome changes will aid the better understanding of mycovirus-fungal interactions.

Although the present study clearly demonstrated that the FgV1-DK21 dsRNA in new hosts can be transmitted to new species via protoplast fusion, it is necessary to test whether virus infected stains can transfer the FgV1-DK21 dsRNA to the same virulent species under field conditions. Therefore, an integrative knowledge including the effect of the timing and rate of application for practical application of the FgV1-DK21 dsRNA is required to establish efficacy and consistency of biological control. In addition, the success of biological control using hypovirulent mycovirus requires a sufficient understanding of the replication mechanism of the mycovirus and also of the interactions between the virus and host fungus, between the virus–fungus and plant, and between the virus–fungus–plant and the rest of the agro-ecosystem. Therefore, efficient and/or practical application of hypovirulent mycoviruses as biological control agents will require more research to elucidate their modes of action at the molecular level and to characterize their ecological fitness.

## Materials and Methods

### Fungal strains and culture conditions

All strains used in this study (Table 1) were stored in 25% (v/v) glycerol at −80°C and were reactivated on potato dextrose agar (PDA; Difco). For total RNA extraction, strains of the *Fg* complex were grown in 50 ml of liquid complete medium (CM) at 25°C at 150 r.p.m. for 5 days while strains of *C. parasitica* were grown in 50 ml of EP complete medium [31] at 26°C and 120 r.p.m. for 5 days. Mycelia were harvested by filtration through Miracloth (Calbiochem) and ground to a fine powder with a mortar and pestle in liquid nitrogen.

### Construction of antibiotic resistant mutants

Protoplasts of fungal strains were prepared by treatment of fresh mycelia grown on YPG liquid medium (0.3% yeast extract, 1% peptone, 2% glucose) for 3 h at 30°C with 1 M NH_4_Cl containing 10 mg/ml of driselase (InterSpex Products), as described previously [32]. Plasmid DNA (20 µg) was directly added along with 1 ml of PEG solution (60% polyethylene glycol 3350, 10 mM Tris-HCl pH 7.5, 10 mM CaCl_2_) to protoplast suspensions. Transformants with resistance to hygromycin B were obtained by transforming the fungal protoplasts with the plasmid pUCH1 [33] and selected for on regeneration medium containing 80 µg/ml of hygromycin B (Calbiochem). For construction of the geneticin-resistant mutant, the plasmid pII99 [34] was transformed into protoplasts of virus-free *F. boothii*. Following the transformation, FgV1-DK21 was transmitted by anastomosis and screened on PDA containing 50 µg/ml of geneticin (Duchefa). For genomic DNA extraction, fungal strains of the *Fg* complex were grown in 50 ml of CM at 25°C, 150 r.p.m. for 5 days. The mycelia were harvested by filtration through sterile Whatman no. 2 filter paper, ground in liquid nitrogen using a mortar and pestle, and then suspended in CTAB buffer [2% CTAB (cetyltrimethyl ammonium bromide), 20 mM EDTA, 0.1 M Tris-HCl, and 1.4 M NaCl]∶2-mercaptoethanol (100∶1). Genomic DNA was extracted sequentially with chloroform∶isoamyl alcohol (24∶1), precipitated with isopropanol. The extracted genomic DNA was extracted twice with phenol∶chloroform∶isoamyl alcohol (25∶24∶1), treated with RNase A (20 µg/ml) for 1 h at 37°C, precipitated with isopropanol, and then finally suspended in distilled water. For the Southern hybridization of hygromycin B-resistant mutants, the extracted genomic DNA was digested with *Kpn* I for 12 h, and the Southern hybridization of G418-resistant mutants, it was digested with *Spe* I and *Kpn* I for 12 h at 37°C. A 10-µl of the digested DNA was separated on 0.8% agarose gel for 8 h. The gel was submerged twice in denaturation solution (1.5 M NaCl and 0.5 N NaOH) for 20 min at room temperature and capillary blotted onto a positively charged nylon transfer membrane (GE Healthcare) in 0.4 N NaOH. Probe labeling reactions were performed in 20 µl of 10 mM Tris-HCl pH 7.5, 7 mM MgCl_2_, 0.1 mM DTT, 30 µCi [α-^32^P] dCTP, 3 mM dNTP mix, 10 pmoles of random primers and 2 U klenow fragment (TaKaRa). After hybridization, unhybridized probe is removed by washing in low stringency wash buffer (2×SSC and 0.1% SDS) and high stringency wash buffer (0.1×SSC and 0.1% SDS). Hybridization signal intensities were measured using a Bio-imaging Analyzer system (BAS-2500; Fuji Film).

### Polymerase chain reaction (PCR) and nucleotide sequencing

PCR of TEF and histone H3 gene region was performed as described with modification [16], [21], [35] using the following conditions: one step at 94°C for 3 min; 35 cycles at 93°C for 45 sec, 55°C for 40 sec, and 72°C for 1 min; and finally one step at 72°C for 10 min. PCR products amplified from fungal strains of the *Fg* complex were extracted from an agarose gel with QIAquick® gel extraction kit (Qiagen) by following the manufacturer's instructions. DNA sequencing was performed at the National Instrumentation Center for Environmental Management of the Seoul National University with an ABI Prism 3730 XL DNA Analyzer (Applied Biosystems) according to manufacturer's instructions. The sequence data were analyzed using a BLAST search tool and were aligned using Clustal W [36].

### Protoplast fusion

Protoplast fusion was performed according to a previously described method with modifications [37]. Young mycelia were prepared as described previously [32], [38] and incubated for 3 h at 30°C with 1 M NH_4_Cl containing 5 mg/ml of driselase and 8 mg/ml of lysing enzyme (L1412; Sigma). Protoplasts were harvested by centrifugation at 2,544× *g* at 4°C for 10 min, washed twice with STC (1.2 M Sorbitol, 10 mM Tris-HCl pH 7.5, 50 mM CaCl_2_), and suspended in 300 µl of MMC buffer (0.6 M Mannitol, 10 mM MOPS pH 7.0, and 10 mM CaCl_2_). Equal volumes of the two protoplast suspensions (100 µl of 1×10^7^ protoplasts/ml) were mixed and placed on ice for 30 min. After 500 µl of PEG solution (60% PEG 3350, 10 mM MOPS pH 7.0, and 10 mM CaCl_2_) was added to the protoplast suspension, the mixture was incubated at 20°C for 20 min. Protoplast fusants were regenerated in 700 µl of potato dextrose broth (PDB; Difco) for 7 days in the dark, plated on 15 ml of YCDA (0.1% yeast extract, 0.1% casein hydrolysate, 0.5% glucose, and 1.5% agar), and then selected on PDA containing 50 µg/ml of hygromycin B and 50 µg/ml of geneticin. Antibiotic-resistant colonies were screened again on hygromycin B-containing PDA.

### RNA extraction and RT-PCR

Total RNA was isolated with extraction buffer according to a previously described method [39] and further treated with DNase I (TaKaRa) to remove genomic DNA. The samples were precipitated with ethanol and finally suspended in DEPC-treated water. To detect viral dsRNA in virus-infected colonies, cDNAs were synthesized with M-MLV reverse transcriptase (Promega) and oligo d(T) primer. The resulting cDNAs (20 ng of input RNA) were used to detect FgV1-DK21 (using primer pairs 5′-TGTGGGAGAAGAAGTATGGCCT-3′ and 5′-ATCAGGAACCATTGAAAGAGTCC-3′ (RdRp region) or 5′-ATGGACACCAAGGATATTTA-3′ and 5′-TTAGGGGTGCAAGGCCCTTTTC-3′ (ORF2 region)). PCR reactions were performed using the following conditions: one step at 94°C for 3 min; 35 cycles at 93°C for 45 sec, 60°C for 40 sec, and 72°C for 1 min 30 sec; and finally one step at 72°C for 10 min. PCR products were analyzed by 1% agarose gel electrophoresis.

### Virulence assays

Virulence assays with *F. boothii*, *F. asiaticum*, and *F. graminearum* were performed as described [40] on wheat cv. Jokyoung. The plants were approximately 6 weeks old and had flowering heads. For production of conidial inoculum, five mycelial plugs were incubated in CMC liquid medium (1.5% carboxymethyl cellulose, 0.1% yeast extract, 0.05% MgSO_4_•7H_2_O, 0.1% NH_4_NO_3_, and 0.1% KH_2_PO_4_) at 25°C and 150 r.p.m. for 5 to 7 days. Conidia were collected by filtering through six layers of sterile cheese cloth. A 10-µl volume of the spore suspension (10^5^ conidia/ml) in 0.01% (v/v) Tween-20 was injected into one floret of each flowering wheat head. Wheat plants inoculated with 0.01% (v/v) Tween-20 alone served as a control. For each treatment, 10 replicate wheat heads were inoculated. Inoculated plants were placed in a growth chamber (25°C, 80% relative humidity, 14/10 h light/dark cycle). Wheat heads were examined for symptoms 14 days post-inoculation.

Virulence of *F. oxysporum* f. sp. *lycopersici* strains was measured with a Fusarium wilt assay as described previously [41]. Ten-day-old tomato seedlings in the four-leaf stage were inoculated by dipping the roots for 3 min in a suspension containing 10^5^ microconidia/ml of the *F. oxysporum* f. sp. *lycopersici* strains in distilled water. Twenty seedlings per treatment were planted in pots containing sterile soil and maintained in a growth chamber at 28°C with 14/10 h light/dark cycle. Severity of disease symptoms was calculated using an index from 0 (healthy plant) to 4 (dead plant).

Virulence of *C. parasitica* strains was assayed as described previously with minor modifications [42]. Mycelial plugs were prepared from the edge of 7-day-old colonies on PDA. Apple tissues (5 mm diameter×5 mm deep) were removed and the insides were filled with mycelial plugs. Following inoculation, they were sealed with plastic wrap to maintain humidity and incubated at 25°C with a 12/12 h light/dark cycle. The discolored area was measured at 14 days post-inoculation. All virulence assays were repeated three times. Statistical analysis was performed with the PASW statistics software (SPSS Inc.).

## Supporting Information

Figure S1 **Alignments of histone H3 (A) and translation elongation factor 1α (B) sequences from** ***F. boothii*** **strains.** Green boxes indicate uniquely fixed nucleotide characters. The following nucleotides are fixed for *F. boothii*: histone H3 positions 148 (C), 252 (C), 300 (T), and 404 (T); translation elongation factor 1α position 616 (C). GenBank accession numbers of nucleotide sequences (histone H3/translation elongation factor 1α) used are as follows: NRRL 26916 (AY452827.1/AF212444.1), NRRL 29011 (AY452828.1/AF212445.1), NRRL 29020 (AY452829.1/AF212443.1), NRRL 29105 (AY452838.1/AF212446.1).
(TIF)

Figure S2 **Detection of FgV1-DK21 RNAs in recipient virus-infected strains.** Total RNAs extracted from *Fusarium graminearum* species complex (A) and *Cryphonectria parasitica* strains (B). Equal amounts (3 µg) of total RNA were electrophoresed through a 0.8% agarose gel in TAE buffer system. *Fb*: *F. boothii*; *Fg*: *F. graminearum*; *Fa*: *F. asiaticum*; *Cp*: *Cryphonectria parasitica*. Lanes 1, 3, 5, and 7 and 2, 4, 6, and 8 represent virus-free and virus-infected strains, respectively in panel A, while in panel B, lanes 1–6 represent virus-free *F. boothii*, virus-infected *F. boothii*, EP155, and four FgV1-DK21-infected strains, respectively. (C) Quantitative RT-PCR analysis of virus-infected strains. Presence of viral dsRNA was confirmed by RT-PCR amplification with a primer pair designed from the RdRp coding region of FgV1-DK21. PCR products were separated on 1% agarose gel. Lanes 1–6 represent negative control (no DNA template), *F. boothii*, *F. graminearum*, F. *asiaticum*, *F. oxyspocum* f. sp. *lycopersici*, and *C. parasitica* (#4), respectively. Lane M denotes 1-kb ladder DNA size marker.
(TIF)

Figure S3 **AFLP fingerprints of genomic DNAs of virus-free and virus-infected fungal strains.** (A) *Fusarium* strains. *Fb*, *F. boothii*; *Fg*, *F. graminearum*; *Fa*, *F. asiaticum*; *Fo*, *F. oxysporum* f. sp. *lycopersici*; Lane M, λ DNA; lanes 1, 3, 5, and 7, virus-free strains; lanes 2, 4, 6, and 8, virus-infected strains. (B) *C. parasitica* (*Cp*) strains. Lane M, λ DNA; lane 1, virus-free *F. boothii*; lane 2, virus-infected *F. boothii*; lane 3, EP155; lane 4, UEP; lanes 5 to 8, EP155 infected with FgV1-DK21. Genomic DNAs of λ DNA and fungal strains were amplified with the primer combinations *Eco*R I +0/*Mse* I +0 and *Eco*R I +CA/*Mse* I +GC, respectively. (+0 indicates no selective nucleotides, +CA and +GC indicate selective nucleotides). The molecular weight size range of the fingerprints is 100–500 nucleotides.
(TIF)

Text S1 **AFLP fingerprints of genomic DNAs of virus-free and virus-infected strains.**
(DOC)
