# Supplementary figures and images for: Transmission of Fusarium boothii Mycovirus via Protoplast Fusion Causes Hypovirulence in Other Phytopathogenic Fungi
Source: PLoS One. 2011 Jun 29;6(6):e21629. doi: 10.1371/journal.pone.0021629 (PMC3126848; doi:10.1371/journal.pone.0021629)

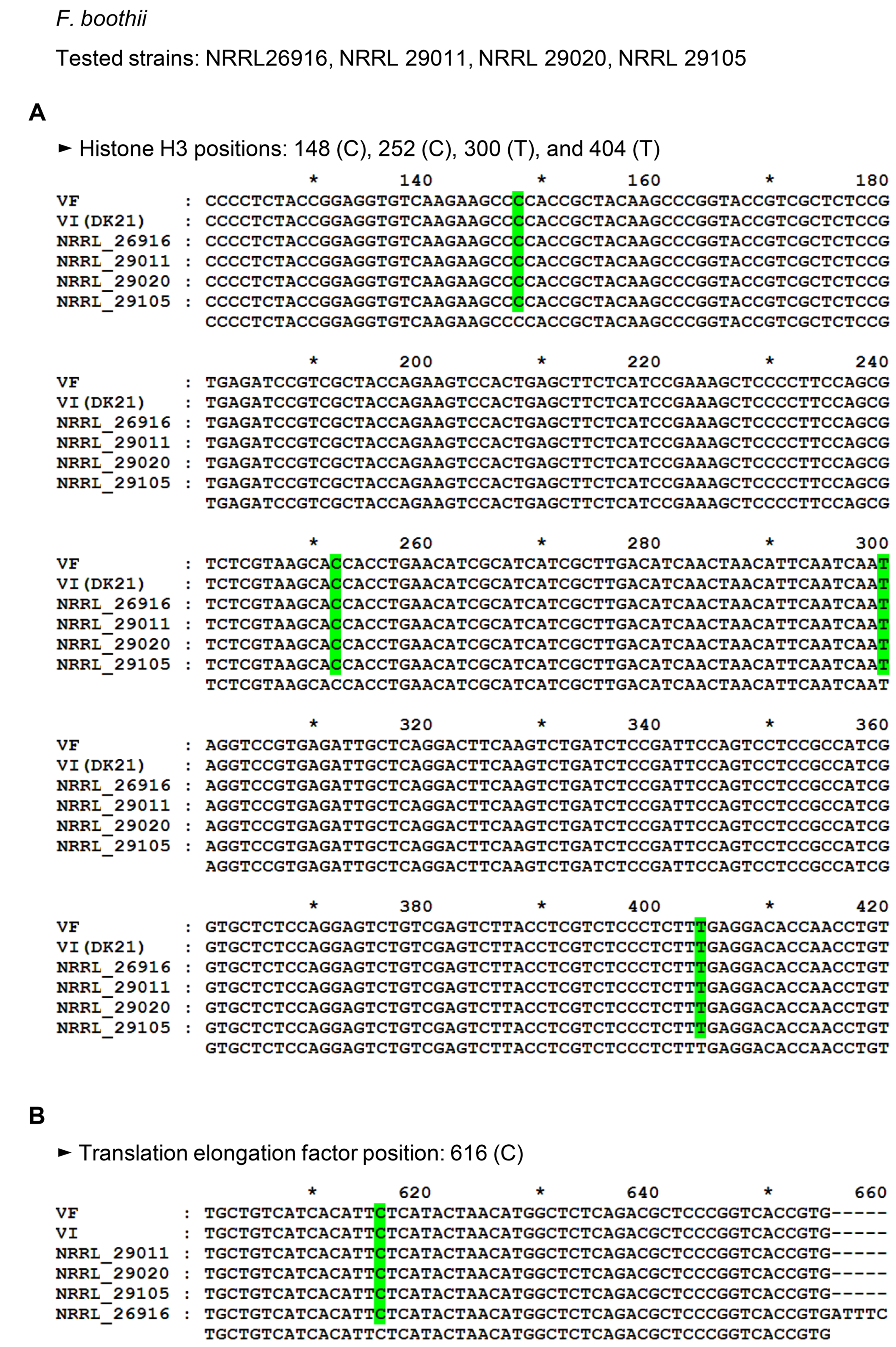

Supplement: Figure S1 — Alignments of histone H3 (A) and translation elongation factor 1α (B) sequences from F. boothii strains. Green boxes indicate uniquely fixed nucleotide characters. The following nucleotides are fixed for F. boothii: histone H3 positions 148 (C), 252 (C), 300 (T), and 404 (T); translation elongation factor 1α position 616 (C). GenBank accession numbers of nucleotide sequences (histone H3/translation elongation factor 1α) used are as follows: NRRL 26916 (AY452827.1/AF212444.1), NRRL 29011 (AY452828.1/AF212445.1), NRRL 29020 (AY452829.1/AF212443.1), NRRL 29105 (AY452838.1/AF212446.1). (TIF) [file pone.0021629.s001.tif]

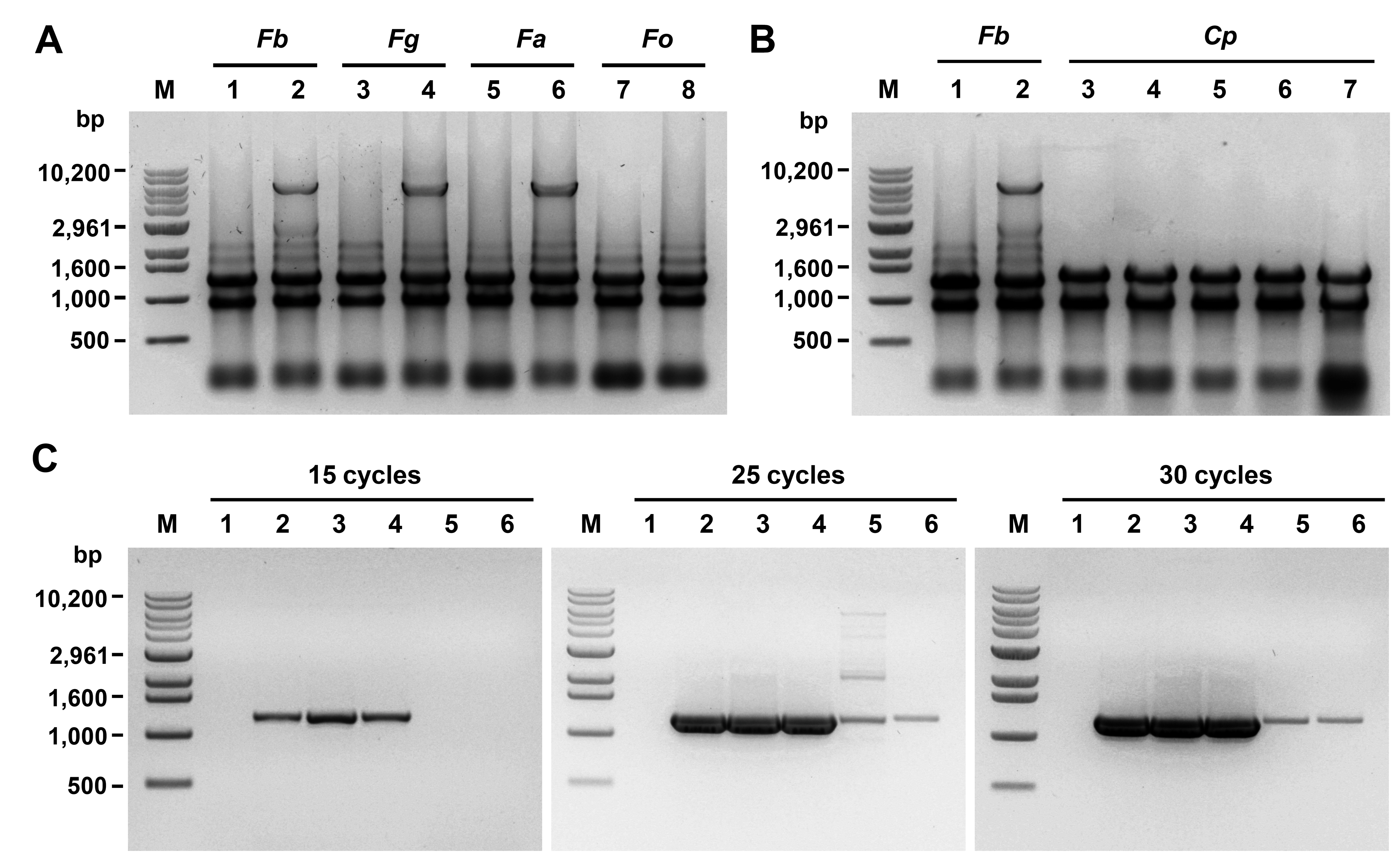

Supplement: Figure S2 — Detection of FgV1-DK21 RNAs in recipient virus-infected strains. Total RNAs extracted from Fusarium graminearum species complex (A) and Cryphonectria parasitica strains (B). Equal amounts (3 µg) of total RNA were electrophoresed through a 0.8% agarose gel in TAE buffer system. Fb: F. boothii; Fg: F. graminearum; Fa: F. asiaticum; Cp: Cryphonectria parasitica. Lanes 1, 3, 5, and 7 and 2, 4, 6, and 8 represent virus-free and virus-infected strains, respectively in panel A, while in panel B, lanes 1–6 represent virus-free F. boothii, virus-infected F. boothii, EP155, and four FgV1-DK21-infected strains, respectively. (C) Quantitative RT-PCR analysis of virus-infected strains. Presence of viral dsRNA was confirmed by RT-PCR amplification with a primer pair designed from the RdRp coding region of FgV1-DK21. PCR products were separated on 1% agarose gel. Lanes 1–6 represent negative control (no DNA template), F. boothii, F. graminearum, F. asiaticum, F. oxyspocum f. sp. lycopersici, and C. parasitica (#4), respectively. Lane M denotes 1-kb ladder DNA size marker. (TIF) [file pone.0021629.s002.tif]

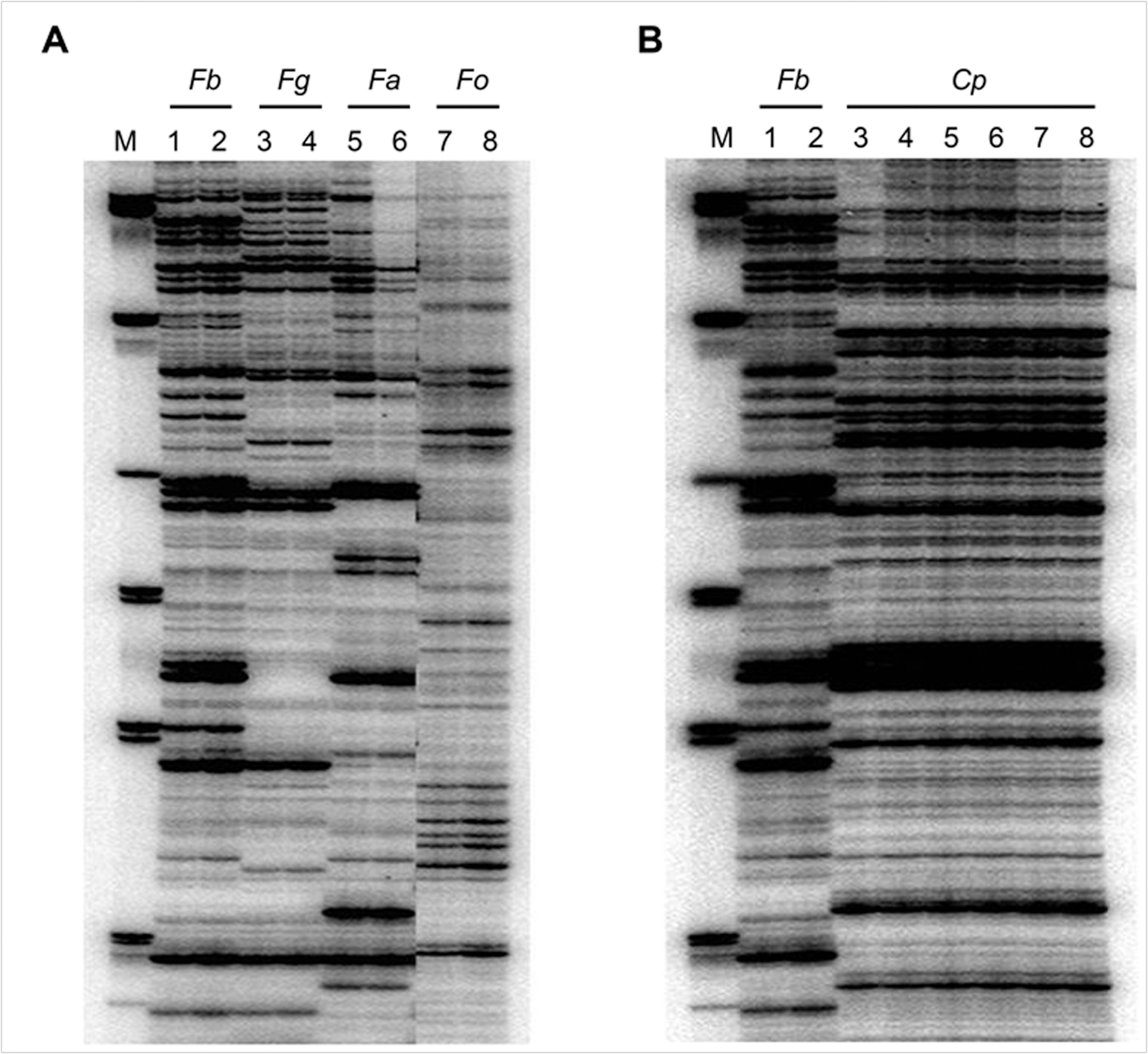

Supplement: Figure S3 — AFLP fingerprints of genomic DNAs of virus-free and virus-infected fungal strains. (A) Fusarium strains. Fb, F. boothii; Fg, F. graminearum; Fa, F. asiaticum; Fo, F. oxysporum f. sp. lycopersici; Lane M, λ DNA; lanes 1, 3, 5, and 7, virus-free strains; lanes 2, 4, 6, and 8, virus-infected strains. (B) C. parasitica (Cp) strains. Lane M, λ DNA; lane 1, virus-free F. boothii; lane 2, virus-infected F. boothii; lane 3, EP155; lane 4, UEP; lanes 5 to 8, EP155 infected with FgV1-DK21. Genomic DNAs of λ DNA and fungal strains were amplified with the primer combinations EcoR I +0/Mse I +0 and EcoR I +CA/Mse I +GC, respectively. (+0 indicates no selective nucleotides, +CA and +GC indicate selective nucleotides). The molecular weight size range of the fingerprints is 100–500 nucleotides. (TIF) [file pone.0021629.s003.tif]
