# Supplementary material for: Transmission of Fusarium boothii Mycovirus via Protoplast Fusion Causes Hypovirulence in Other Phytopathogenic Fungi
Source: PLoS One. 2011 Jun 29;6(6):e21629. doi: 10.1371/journal.pone.0021629 (PMC3126848; doi:10.1371/journal.pone.0021629)
Supplement: Text S1 — AFLP fingerprints of genomic DNAs of virus-free and virus-infected strains. (DOC) [file pone.0021629.s004.doc]

**Materials and Methods**

**AFLP fingerprints of genomic DNAs of virus-free and virus-infected strains.**

The genomic DNA extraction and AFLP analysis were perfomed as previously described . Genomic DNA (500 ng) was digested with 5 U *Eco*RⅠ and 5 U *Mse*Ⅰ in 40 μl mixtures containing 20 mM Tris-acetate, 10 mM magnesium acetate, 50 mM potassium acetate, 1 mM DTT, and 50 ng/μl BSA for 3 h at 37^o^C. Then 10 μl of a mixture containing 5 pmoles of *Eco*RⅠ adapters (5′-CTCGTAGACTGCGTACC-3′ and 5′-AATTGGTACGCAGTCTAC-3′), 50 pmoles of *Mse*Ⅰ adapters (5′-GACGATGAGTCCTGAG-3′ and 5′-TACTCAGGACTCAT-3′), 20 mM Tris-acetate, 10 mM magnesium acetate, 50 mM potassium acetate, 10 mM DTT, 1 mM ATP, 30 mM Tris-HCl pH 7.8, 10 mM MgCl_2_, 50 ng/μl BSA, and 1 U T4 DNA ligase were added, and the ligation reaction was incubated for 4 h at 16^o^C. Following ligation, the reaction mixture was diluted 10-fold with 10 mM Tris-HCl and 0.1 mM EDTA at pH 8.0 and stored at -20^o^C. A 5-μl volume of this mixture was used as template for PCR with 20 μl of reaction mixture. The PCR was primed with oligomers having no or two selective nucleotides. The *Eco*RⅠprimers were labeled at their 5′-end with 100 μCi [γ-^32^P] ATP and 10 U T4 polynucleotide kinase (TaKaRa). The PCR products were mixed with an equal volume of loading dye (98% deionized formamide, 10 mM EDTA, 0.03% bromphenol blue, and xylene cyanol FF). The mixture was denatured at 90^o^C for 3 min and immediately placed on ice. Aliquots (2 μl) of each reaction were electrophoresed through 6% sequencing gel at 60 W for ~3 h . Gels were fixed for 30 min in 10% acetic acid and 10% ethanol. After the gels were dried, fingerprint images were visualized using a Fuji BAS-2500 Phosphor Imager and the corresponding Imaging software (Fuji Photo Film Company Ltd).

1. Vos P, Hogers R, Bleeker M, Reijans M, van de Lee T, et al. (1995) AFLP: a new technique for DNA fingerprinting. Nucleic Acids Res 23: 4407-4414.

2. Churchill ACL, Ciuffetti LM, Hansen DR, Van Etten HD, Van Alfen NK (1990) Transformation of the fungal pathogen *Cryphonectria parasitica* with a variety of heterologous plasmids. Curr Genet 17: 25-31.

3. Kerenyi Z, Zeller K, Hornok L, Leslie JF (1999) Molecular standardization of mating type terminology in the *Gibberella fujikuroi* species complex. Appl Environ Microbiol 65: 4071-4076.

4. Leslie JF, Summerell BA (2006) The *Fusarium* laboratory manual. Ames: Blackwell Publishing. 67 p.
